# A novel structurally identified epitope delivered by macrophage membrane-coated PLGA nanoparticles elicits protection against *Pseudomonas aeruginosa*

**DOI:** 10.1186/s12951-022-01725-x

**Published:** 2022-12-14

**Authors:** Chen Gao, Yin Chen, Xin Cheng, Yi Zhang, Yueyue Zhang, Ying Wang, Zhiyuan Cui, Yaling Liao, Ping Luo, Weihui Wu, Cheng Wang, Hao Zeng, Quanming Zou, Jiang Gu

**Affiliations:** 1 National Engineering Research Center of Immunological Products, Department of Microbiology and Biochemical Pharmacy, College of Pharmacy, Army Medical University, The 30th, Gaotanyan Street, Shapingba District, Chongqing, 400038 People’s Republic of China; 2 State Key Laboratory of Trauma, Burns and Combined Injury, Institute of Combined Injury of PLA, Chongqing, Engineering Research Center for Nanomedicine, College of Preventive Medicine, Army Medical University, Chongqing, 400038 China; 3 953Th Hospital, Shigatse Branch, Xinqiao Hospital, Army Medical University, (Third Military Medical University), Shigatse, 857000 China; 4 State Key Laboratory of Medicinal Chemical Biology, Key Laboratory of Molecular Microbiology and Technology of the Ministry of Education, Department of Microbiology, College of Life Sciences, Nankai University, Tianjin, China

**Keywords:** Outer membrane protein, Epitope-based vaccine, Nanoparticle, *Pseudomonas aeruginosa*

## Abstract

The increasing prevalence of antibiotic resistance by *Pseudomonas aeruginosa* (PA) raises an urgent need for an effective vaccine. The outer membrane proteins of PA, especially those that are upregulated during infection, are ideal vaccine targets. However, the strong hydrophobicity of these proteins hinders their application for this purpose. In this study, we selected eight outer membrane proteins from PA with the most significantly upregulated expression. Their extracellular loops were analyzed and screened by using sera from patients who had recovered from PA infection. As a result, a novel immunogenic epitope (Ep_167-193_) from PilY1 (PA4554) was found. Moreover, we constructed a macrophage membrane-coated PLGA (poly lactic-co-glycolic acid) nanoparticle vaccine carrying PilY1 Ep_167-193_ (PNPs@M-Ep_167-193_) that elicits a Th2 immune response and confers adequate protection in mice. Our data furnished the promising vaccine candidate PNPs@M-Ep_167-193_ while providing additional evidence for structure-based epitope identification and vaccine design.

**Graphical Abstract:**

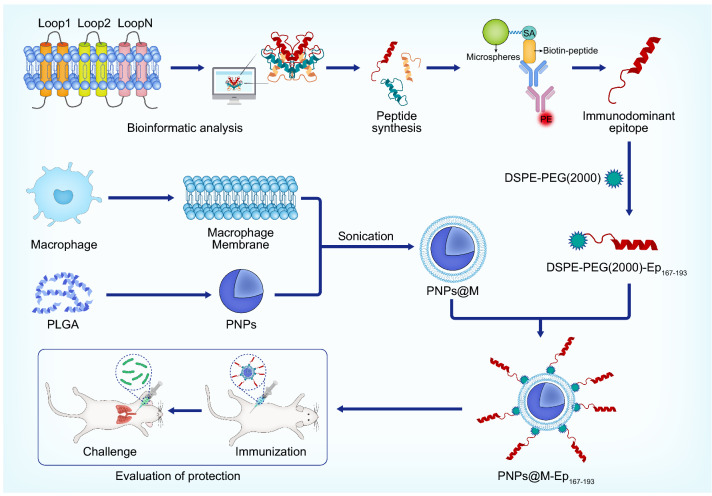

**Supplementary Information:**

The online version contains supplementary material available at 10.1186/s12951-022-01725-x.

## Introduction

*Pseudomonas aeruginosa* (PA) is one of the common pathogens that causes hospital-acquired infections [1]. In particular, patients with impaired respiratory tracts are at high risk for PA infection, such as patients with mechanical ventilation, COPD (chronic obstructive pulmonary disease), bronchiectasis or cystic fibrosis [2, 3]. Additionally, PA is the primary bacterial pathogen responsible for infection after burns and trauma [4, 5]. In recent years, the antibiotic resistance of PA has gradually increased, which has led to the development of pan-drug resistant and multi-drug resistant PA strains [3]. Thus, controlling PA infection and antibiotic resistance has become a severe public health problem. The effectiveness of conventional antibiotic therapy is becoming increasingly limited, which promotes an urgent need for alternative strategies [6]. Indeed, PA vaccines can prevent the occurrence of infectious diseases and reduce the use of antibiotics to ultimately curb the appearance of drug resistance. However, no PA vaccine has yet been successfully marketed [7].

Proteins or polysaccharides (such as capsular polysaccharides or lipopolysaccharides) on bacterial membranes are the main targets of vaccine antigens against bacteria. This is because antibodies against these antigens not only mediate antibody-dependent cell-mediated cytotoxicity (ADCC) effects but also block their pathogenic effects. For example, antibodies targeting capsular polysaccharides are able to inhibit the antiphagocytic effect mediated by the capsular polysaccharides of *Streptococcus pneumoniae *[8]*.* PA swimming mediated by the bacterial flagellum was blocked by anti-flagellum antibodies [9]. Many bacterial outer membrane proteins have been investigated as vaccine candidates, such as OprF of PA [10], fHBP (factor H binding protein) of group B *Neisseria meningitidis* [11], and intimin of *E. coli* O157 [12]. However, the strong hydrophobicity of these outer membrane proteins is the greatest challenge encountered when they are used as vaccine candidates.

Recent advances in structural vaccinology have provided solutions to the challenge of using outer membrane proteins as vaccine candidates [13]. Most outer membrane proteins consist of transmembrane regions and extracellular loops, of which the loops are the central functional and immunogenic regions [14]. In a preliminary study guided by the structure of the outer membrane protein A (OmpA) from *E. coli* K1, we found that all four loops of OmpA are immunogenic [15]. Building on these findings, we constructed a promising vaccine candidate, Vo [16]. Additionally, we speculate that this structure-based vaccine design could also be applied to the development of vaccines targeting the outer membrane proteins of PA.

In our previous study, after infecting mice with PA, the changes in the PA transcriptome were characterized. The upregulated protein OprH was identified and showed good immunogenicity [17]. According to the transcriptome data, eight outer membrane proteins with the most significantly upregulated expression were selected in this study. The extracellular loops of these proteins were analyzed with informatics tools. The B-cell epitopes with good immunogenicity were screened with a Luminex-based method using sera from patients who had recovered from PA infection. A macrophage membrane-encapsulated PLGA nanoparticle was constructed as a vehicle to deliver the identified B-cell epitopes. Furthermore, the immune response and protective effect of the nanovaccine were evaluated in a mouse model.

## Materials and methods

### Mice, strains and serum samples

Six- to eight-week-old specific pathogen-free female BALB/c mice were purchased from Beijing Vital River Laboratory Animal Technology Company Limited (Beijing, China). PA XN-1 (CCTCC M2015730) was isolated from the sputum of a patient with severe pneumonia at Southwest Hospital in Chongqing, China [18]. Sera were collected from PA-infected convalescent patients and healthy donors. Written informed consent forms (ICFs) were collected from all participants. All animal care and experiments complied with ethical regulations and were approved by the Animal Ethical and Experimental Committee of the Third Military Medical University (No. AMUWEC2020967).

### Screening immune-dominant epitopes

After evaluation of the transcriptome results, eight transmembrane proteins with the most significant changes in mRNA levels were selected. The protein information is listed in Additional file 1: Table S1. The structures of PA1777, PA4067, PA0595, PA0958 and PA2398 are from the PDB database, while the structures of PA1178, PA4554 and PA0165 were modeled by SWISS-MODEL and validated by Procheck and QMEAN [19, 20]. Then, PRED-TMBB (http://bioinformatics.biol.uoa.gr/PRED-TMBB/input.jsp) was used to predict the extracellular loops, transmembrane domains and intercellular loops [21]. A total of 54 peptides corresponding to the putative extracellular loop were synthesized and labeled with biotin (Additional file 2: Table S2). Ten serum samples were collected from PA-infected convalescent patients and another ten serum samples were collected from healthy donors. (See Additional file 3: Table S3).

A Luminex-based assay was set up to screen for dominant epitopes. Briefly, 30 μg of streptavidin (Thermo Fisher, Waltham, US) was covalently coupled to 2.5×10^6^ beads according to the manufacturer’s instructions (Luminex, Austin, US). The beads were then incubated with biotin-tagged peptides (2 μg/ml) at 37 °C for 1 h. After washing with phosphate-buffered saline plus 0.1% Tween-20 (PBST), serum samples diluted 1:200 were added and incubated at 37 °C for 1 h. After the removal of the supernatant, the beads were washed with PBST. A phycoerythrin (PE)-labeled goat anti-human secondary antibody (Abcam, Cambridge, UK) at a 1:2500 dilution was added for incubation at 37 °C for 40 min. Finally, the fluorescence intensity of the beads was measured using a Luminex 200 instrument, and the results are expressed as the median fluorescence intensity (MFI). The MFI of beads without peptides was used as a control. Another hundred serum samples from patients recovered from PA infection were collected and used to verify the top 10 dominant epitopes. The methods were the same as those described above.

To evaluate the immunogenicity of the eight peptides (PA4554 D148-T172, PA4554 C167-W193, PA0958 A200-Q235, PA2398 T302-V331, PA2398 F636-G672, PA2398 K694-Q712, PA2398 Q737-K754, PA0165 R163-A174) in mice, these peptides were synthesized and conjugated to KLH (keyhole limpet hemocyanin) (Sigma, Milwaukee, US). The peptide–KLH conjugates formulated with the adjuvant Al(OH)_3_ were used for intramuscular immunization of BALB/c mice on day 0, day 14 and day 21. The injection volume for each animal was 500 μl, and each injection contained 100 μg of the conjugated preparation and 500 µg of Al(OH)_3._ PBS and Al(OH)_3_ were used as controls. Blood was collected via the tail vein seven days after the final immunization, and serum was isolated and stored at − 80 °C.

### ELISA

The reactivity of mouse sera against each peptide was determined by ELISA. The 96-well ELISA plates (Costar) were precoated with streptavidin (2 μg/ml) overnight at 4 °C. Blocking was performed with 1% bovine serum albumin (BSA) in PBST. Then, 2 μg/ml biotin-tagged peptide was added and incubated at 37 °C for 1 h. After the plates were washed with PBST, diluted serum samples (starting a dilution of 1:100 followed by 2-fold serial dilutions) were added for incubation at 37 °C for 1 h. Then, HRP-labeled goat anti-mouse IgG (Abcam) was added at a 1:5000 dilution for incubation at 37 °C for 45 min. The color was developed with TMB substrate solution (Beijing ZSGB-BIO) after washing, and the absorbance was measured at 450 nm. A sample was considered positive when the measured absorption value was more than 2.1-fold greater than the negative control (preimmune).

### Preparation of PNPs, PNPs@M, and PNPs@M-Ep_167-193_

The PNPs (PLGA nanoparticles) were synthesized via a reported water-in-oil-in-water double emulsion method with slight modifications [22]. In brief, PLGA (poly lactic-co-glycolic acid) (100 mg) was directly dissolved in methylene chloride (2 ml). Subsequently, the mixture was emulsified by sonication (35% amplitude, 2 min) using a Digital Sonifier S-250D (Branson Ultrasonic, Danbury, CT, US) in an ice bath. Next, the primary emulsion was immediately added to 10 ml of PVA (Polyvinyl alcohol) solution (3%, w/v) and sonicated for 3 min to form a double emulsion. The double emulsion was stirred overnight to remove the organic solvent. Then, the product was centrifuged at 12,000 rpm for 15 min and washed three times with deionized water.

Macrophage cell membrane encapsulate was prepared as described previously [23]. The mouse macrophage cells (RAW264.7) were digested with trypsin, frozen at − 80 °C and thawed at room temperature. By repeated freeze–thaw three times, the membrane was collected by centrifugation at 14,000 rpm for 15 min, washed with PBS containing protease inhibitor and sonicated for 5 min. Subsequently, PNPs were mixed with the macrophage cell membrane (1:1 weight ratio of nanoparticles: membrane protein) [23]. The mixture was sonicated in an ice bath for 3 min and maintained at 4 °C overnight. The PNPs@M (Macrophage membrane-coated PLGA nanoparticles) was finally collected by high-speed centrifugation at 12,000 rpm for 15 min. To obtain PNPs@M carrying Ep_167-193_ (PNPs@M-Ep_167-193_), DSPE-PEG (2000)-Ep_167-193_ was dissolved in disinfected water and mixed with the solution of PNPs@M. The mixture was reacted at room temperature for 1 h. The residual DSPE-PEG(2000)-Ep_167-193_ was eliminated by centrifugation at 12,000 rpm for 15 min.

### Characterization analysis

The size and morphology of the nanoparticles were determined using a transmission electron microscope (Tecnai G2 F20 U-TWIN, FEI, Hillsboro, OR, US). The zeta-potential and size distribution were measured at room temperature using a Nano-ZS (Malvern, Worcestershire,UK). To confirm the membrane camouflage, PNPs@M was denatured and resolved via 12% SDS-PAGE. The gel was disassembled and proteins in the gel were stained for 1 to 2 h in Coomassie blue staining solution. Then the gel was destained with 10% acetic acid, which was changed every 30 min until the background is clear [24].

### Toxicity assay

The toxicities of PNPs@M-Ep_167-193_ and PNPs@M on DC2.4 mouse dendritic cells and L929 mouse fibroblast cells were determined by the standard Cell Count Kit (CCK-8) assay. The cells were incubated with PNPs@M-Ep_167-193_ and PNPs@M at various concentrations (0, 25, 50, 100 and 200 μg/ml) for 24 h, 48 h and 72 h, respectively. Erythrocytes (300 μL) diluted in 0.9% NaCl solution were incubated with 1.2 ml of PNPs@M-Ep_167-193_ at 37 °C for 2 h. The absorbance of the supernatant was tested at 450 nm using a microplate reader. The experiments were conducted in triplicate and repeated twice. The biocompatibility of PNPs@M-Ep_167-193_ in vivo was assessed by a mouse experiment. The mice were cared for and treated as demonstrated in the preparation of PNPs@M-Ep_167-193_. On day 0, day 14 and day 21, the mice were immunized intramuscularly with 50 μg of PNPs@M-Ep_167-193_ (based on the concentration of Ep_167-193_)_._ The mice were sacrificed 14 days after the third immunization, and their major organs were obtained by surgery. The pathological changes were observed with an Olympus DX51 optical microscope (Tokyo, JPN) after HE staining. The body temperature and body weight of the mice were monitored and recorded every day during the 35 days of observation.

### Evaluation of the immunogenicity of PNPs@M-Ep_167-193_

A total of 20 BALB/c mice were randomly divided into four groups. On day 0, 14 and 21, the mice in each group were immunized with PNPs@M-Ep_167-193_ (50 μg)_,_ Ep_167-193_ (50 μg)_,_ PNPs@M (50 μg) or PBS. Seven days after the first, second and final immunization, blood was collected via the tail vein, and serum was isolated. The titers of total IgG and the subtypes against Ep_167-193_ in the sera were determined by ELISA as described above. HRP-labeled goat anti-mouse IgG, IgG1, IgG2a or IgG2b (Abcam) at a 1:5000 dilution was used as the secondary antibody.

### Evaluation of protection conferred by immunization with PNPs@M-Ep_167-193_

A total of 15 mice were immunized with PBS, PNPs@M, Ep_167-193_ or PNPs@M-Ep_167-193_ as described above. Seven days after the last immunization, the mice in each group were intratracheally injected with a sublethal dose of PA XN-1 (1.3 × 10^6^ CFU/mouse). Then, the infection was scored according to the breathing, piloerection, movement, nasal secretion and posture of the mice as described previously [25]. The global score was recorded as unaffected (0–1), slightly affected (2–4), moderately affected (5–7), or severely affected (8–10). Mouse body weights were recorded every 24 h for 10 days.

The lungs of the mice were collected 24 h after the challenge and homogenized in 1 ml of sterilized PBS. Homogenates were serially diluted, plated on LB agar plates, and incubated overnight at 37 °C. Counts of viable PA XN-1 were determined by counting the colonies on the agar plates. Additionally, homogenates collected as described above were centrifuged, and the supernatants were used for cytokine analysis. The levels of TNF-α, IL-1β, IL-6 and IL-12 were measured using a Mouse ELISA Kit (Dakewei) according to the manufacturer’s instructions.

Twenty-four hours after the sublethal challenge, the lungs from mice in different groups were collected and fixed with 4% paraformaldehyde. Then, the samples were paraffin-embedded and cut into 4 μm section slices. The slices were stained with hematoxylin and eosin (HE) and viewed by light microscopy at 400×magnification. Each section was given disease scores in terms of the states of hemorrhage, edema, hyperemia, neutrophil infiltration, and destruction of bronchi structure by a pathologist in a blinded fashion according to a previously reported method [25]. Each state was scored from 0 to 2 (0 = none, 2 = severe), and the final score of each section was the sum of the scores from the five states.

### Statistical analysis

Data are shown as the mean ± standard error (SE). The significance of the differences was determined by unpaired parametric test (Student’s t test for two groups or one-way ANOVA for more than three groups). Bacterial burden was analyzed by the nonparametric Mann–Whitney test. IBM SPSS Statistics version 19.0 software (IBM Corp., Armonk, US) and Prism 8.0 software (GraphPad, US) were used to analyze the data. Significance was accepted when *P* < 0.05.

## Results

### Ep_167-193_ from PA4554 is a novel predominant epitope in human and mice

**Fig. 1:**
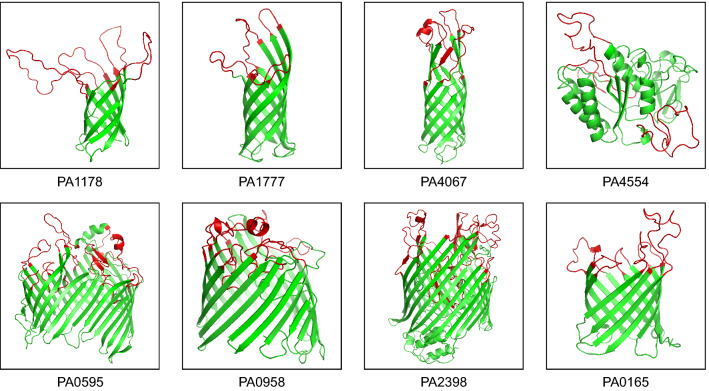
Cartoon images of the 3D structures of the eight transmembrane proteins. The structures of PA1777 (PDB: 4RLC), PA4067 (PDB: 2N6L), PA0595 (PDB: 5IVA), PA0958 (PDB: 2ODJ) and PA2398 (PDB: 1XKH) are from the PDB database, while the structures of PA1178, PA4554 and PA0165 were modeled by SWISS-MODEL. The predicted extracellular loops are highlighted in red

In our preliminary study, the changes in the mRNA expression levels of PA genes during infection were recorded [17]. In this study, eight transmembrane proteins with the most significant changes in mRNA levels were selected. They are PA1178, PA1777, PA4067, PA4554, PA0595, PA0958, PA2398 and PA0165. Their level of mRNA change and genetic information are listed in Additional file 1: Table S1. Using PRED-TMBB software [26], we then analyzed the transmembrane loops of these proteins (Additional file 4: Fig. S1), and the locations of these transmembrane loops are displayed in the crystal structures of the corresponding full-length proteins (Fig. 1). Since the crystal structures of PA1178, PA4554 and PA0165 were not available, these structures were predicted using SWISS-MODEL [27]. Then the predicted structure was validated by Procheck and QMEAN [19, 20]. Results showed that the proportion of residues which occupied the most favored regions for PA1178, PA4554, PA0595 and PA0165 was 92.9%, 93.53% and 85.20%, respectively. The QMEAN Z- scores of the three models lied within the expected range (Additional file 5: Fig. S2). These data demonstrated that the predicted structures of PA1178, PA4554 and PA0165 were acceptable.

**Fig. 2:**
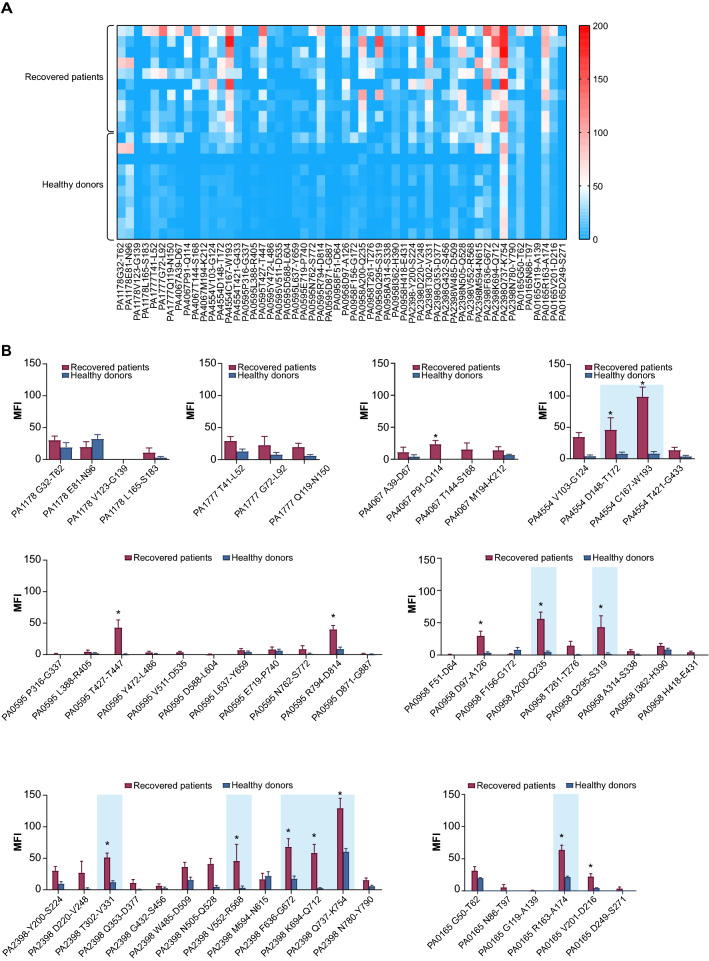
Screening for immunogenic epitopes in serum samples provided by patients who had recovered from PA infection. **A**. Heatmap of the reactivities of 54 peptides (corresponding to those in the predicted extracellular loops) to the sera from 10 recovered patients and 10 healthy donors. **B**. The responses of the peptides to the serum samples clustered according to the individual transmembrane proteins. The bar represents the median fluorescence intensity (MFI) of each peptide determined using the Luminex assay. The top 10 most immunoreactive peptides are labeled with a blue background. Multiple comparisons among recovered patients and healthy donors in each group were analyzed by two-way ANOVA. *P < 0.05

First, a total of 54 peptides spanning the transmembrane loops of the eight proteins were synthesized (Additional file 2: Table S2). Ten serum samples from recovered patients and another ten serum samples from healthy donors were then collected and used to screen the immunogenic epitopes. The pattern of serum IgG binding to the 54 peptide features is shown in the heatmap in Fig. 2A. The responses of the peptides to the sera were clustered by protein and are shown in Fig. 2B. Clearly, in PA1178, the response to peptide L165-S183 was significantly different between the two groups. Nonetheless, no significant difference was observed for the other three peptides. For PA1777, PA4067, PA4554, PA0595, PA0958, PA2398 and PA0165, all of the selected peptides showed an increased response in the sera from the recovered patients. Interestingly, a clear response was detected between PA2398 Q737-K754, PA1178 E81-N96, PA2398 M594-N615 and PA0165 R163-A174 and sera from healthy donors.

**Fig. 3:**
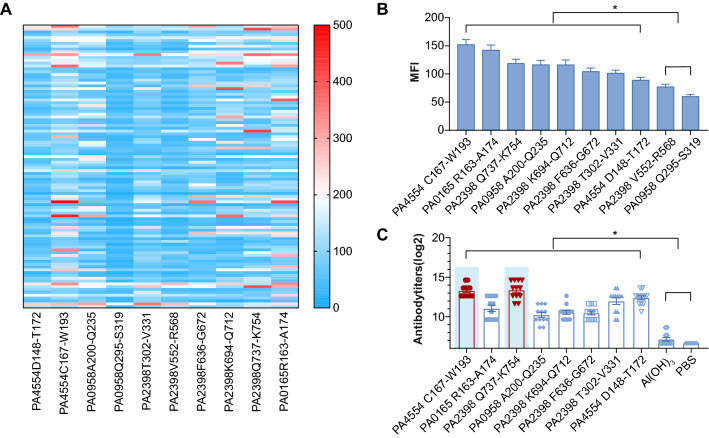
Ep_167-193_ from PA4554 is a novel predominant epitope that shows strong immunogenicity in mice. **A**. Heatmap of ten peptides showing their reactivity to sera from 100 patients who had recovered from PA infection. **B**. Summary of the immunoreactivity results of each peptide. The bar represents the median fluorescence intensity (MFI) of each peptide as determined by the Luminex assay. Multiple comparisons among the groups were analyzed by one-way ANOVA. *P < 0.05. **C**. The bars represent the titers of anti-peptide antibodies in sera from mice immunized with the corresponding peptide conjugated to the KLH carrier protein (n = 10). The top two most immunogenic peptides, PA2398 Q737-K754 and PA4554 C167-W193, are labeled with a blue background. Multiple comparisons among the groups were analyzed by one-way ANOVA. *P < 0.05

The top ten most reactive epitopes were chosen, namely, PA2398 Q737-K754, followed by PA4554 C167-W193, PA2398 F636-G672, PA0165 R163-A174, PA2398 K694-Q712, PA0958 A200-Q235, PA2398 T302-V331, PA4554 D148-T172, PA2398 V552-R568 and PA0958 Q295-S319. Their immunoreactivities were then validated by an additional 100 serum samples from recovered patients. The heatmap in Fig. 3A shows the responses of the ten peptides to the individual serum samples. The summarized results are shown in Fig. 3B. Clearly, the ten selected epitopes showed considerably strong immune reactivities. Next, we evaluated the immunogenicity of the eight peptides in mice with the exception of PA2398 V552-R568 and PA0958 Q295-S319 due to their low immunoreactivities (Fig. 3B). As expected, all tested epitopes induced a significant increase in antibodies when compared with PBS and Al(OH)_3_ treatment (Fig. 3C). Consistent with previous findings [28], PA2398 Q737-K754 was found to be the most immunogenic epitope, followed by PA4554 C167-W193, PA4554 D148-T172, PA2398 T302-V331, PA0165 R163-A174, PA2398 K694-Q712, PA2398 F636-G672 and PA0958 A200-Q235. These data suggest that the eight epitope peptides had good immunogenicity (Fig. 3C).

### Nanoparticles carrying the epitope PA4554 C167-W193 (PNPs@M-Ep_167-193_) were successfully produced

**Fig. 4:**
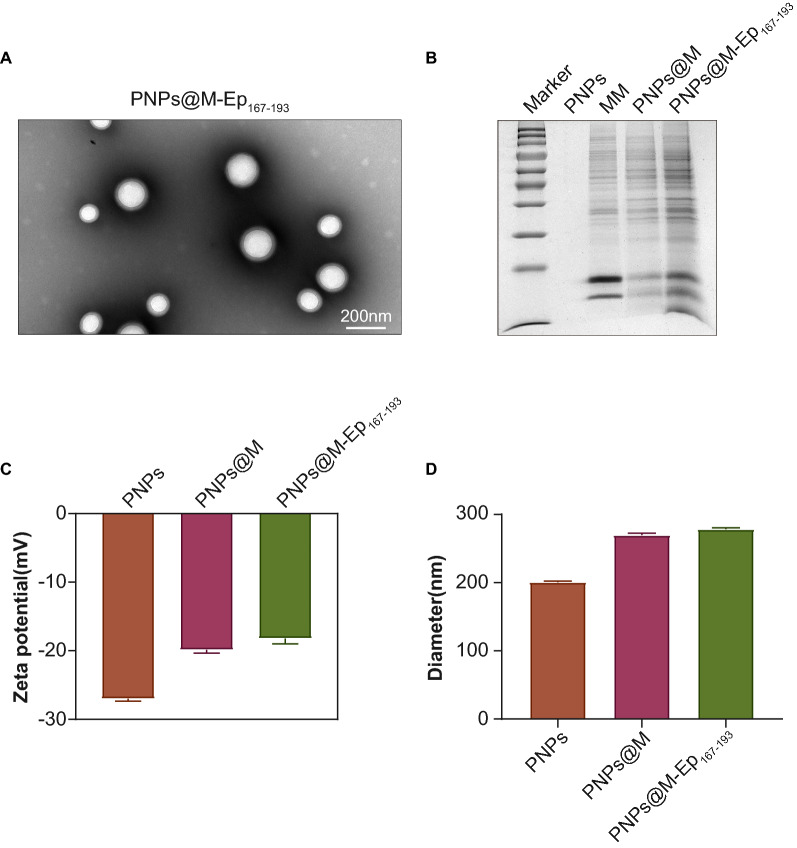
Characterization of PNPs@M-Ep_167-193_. A. Transmission electron microscopy (TEM) image of PNPs@M-Ep_167-193_. The scale is 200 nm. B. SDS–PAGE analysis. The macrophage membrane (MM) protein (20 μg) was resolved by 12% SDS–PAGE. C. Surface charge determination. The results are shown as the mean ± SE. D. DLS determination of the hydrodynamic diameters. The results are shown as the mean ± SE

As noted above, PA2398 Q737-K754 and PA4554 C167-W193 were the top 2 immunogenic epitopes in both humans and mice. The protective effect of PA2398 Q737-K754 was assessed previously. As a result, we investigated the epitope PA4554 C167-W193 (Ep_167-193_) in subsequent studies. Macrophage membrane (MM)-based nanocarriers are known as instrumental vaccine delivery systems that improve the efficacy of immunodominant epitopes in vivo [29]. To enhance the immunogenicity of Ep_167-193_, we fabricated MM-coated PLGA nanoparticles (PNPs@M) to deliver Ep_167-193_, obtaining a functional nanomaterial termed PNPs@M-Ep_167-193_. PNPs were prepared using a double emulsion method. MMs and PNPs were applied to fabricate PNPs@M at the optimum mass ratio of 1:1 (Additional file 6: Fig. S3). SDS–PAGE confirmed the successful coating of the MM (Fig. 4B). The zeta potential determination showed that the MM coating increased the surface charge of the PNPs (averagely − 27 mV) to approximately − 19.8 mV (Fig. 4C). Additionally, the hydrodynamic diameter of the PNPs@M (269 nm, PDI = 0.171) increased when compared with the blank PNPs (Fig. 4D, 201 nm, PDI = 0.028), which was consistent with the effect of platelet membrane cloaking on the PNPs encapsulating indocyanine green [30].

Next, PNPs@M and Ep_167-193_ conjugated to 1,2-distearoyl-sn-glycero-3- -phosphoethanolamine-poly(ethylene glycol)-2000 (DSPE-PEG2000) were employed to prepare PNPs@M-Ep_167-193_ based on the interaction between DSPE segments and the cell membrane [31]. The NPs collected by high-speed centrifugation were spherical with good dispersion ability. Transmission electron microscopy (TEM) showed that the size of PNPs@M-Ep_167-193_ was approximately 242 nm (Fig. 4A). Due to the cationic properties of Ep_167-193_, peptide loading increased the surface charge of PNPs@M slightly (Fig. 4C). Nevertheless, the hydrodynamic diameter of PNPs@M-Ep_167-193_ (272 nm, PDI = 0.281) measured by dynamic light scattering (DLS) was unchanged compared with that of the PNPs@M (Fig. 4D, 269 nm, PDI = 0.171).

### PNPs@M-Ep_167-193_ was safe for in vitro and in vivo evaluation

**Fig. 5:**
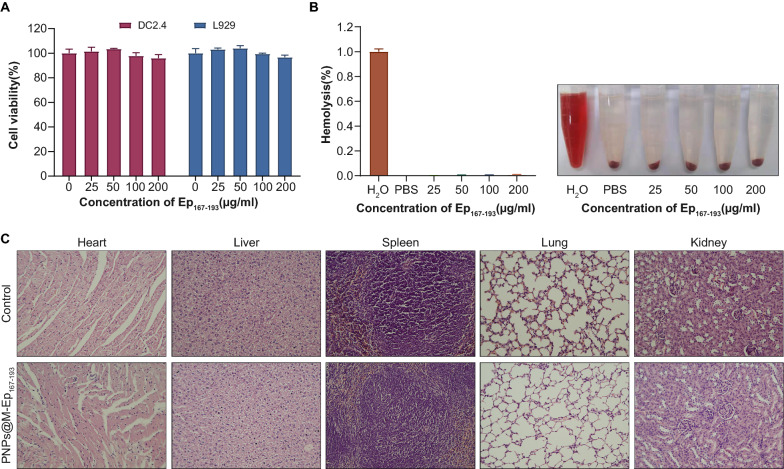
Toxicity evaluation of PNPs@M-Ep_167-193_. **A**. The viability of DC2.4 cells and L929 cells after exposure to different concentrations of PNPs@M-Ep_167-193_ for 24 h. The results are shown as the mean ± SE. **B**. Statistical data (left) and image (right) showing hemolysis induced by different concentrations of PNPs@M-Ep_167-193_. **C**. Hematoxylin and eosin (HE) staining of the organs of mice treated with NaCl and PNPs@M-Ep_167-193_

To evaluate the toxicity of the material, we incubated PNPs@M-Ep_167-193_ with DC2.4 cells and L929 cells at 37 °C for 24 h, 48 h and 72 h, respectively. Notably, the PNPs@M-Ep_167-193_ did not affect cell survival at concentrations up to 200 μg/ml (based on the concentration of Ep_167-193_) at the three observation points (Fig. 5A and Additional file 7: Fig. S4A, B). Meanwhile, the carrier PNPs@M alone was also safe (Additional file 7: Fig.S4C–E). The nanomaterial had a marginal effect on erythrocytes, indicative of nontoxicity in vitro (Fig. 5B). Animal experiments found that three intramuscular injections of 50 μg of PNPs@M-Ep_167-193_ were non-lethal to BALB/c mice. In addition, no significant change of body weight (Additional file 8: Fig. S5A) or temperature fluctuation (Additional file 8: Fig. S5B) in mice was noted during the 35 days of observation (*P* < 0.05). The hearts, livers, spleens, lungs, and kidneys of the treated mice were obtained 14 days after the third immunization. Consistent with the biocompatibility of other cell membrane-based NPs [32], no pathological changes were observed in the abovementioned tissues as revealed by hematoxylin and eosin (HE) staining (Fig. 5C).

### PNPs@M-Ep_167-193_ induced a Th2-biased immune response in mice

**Fig. 6:**
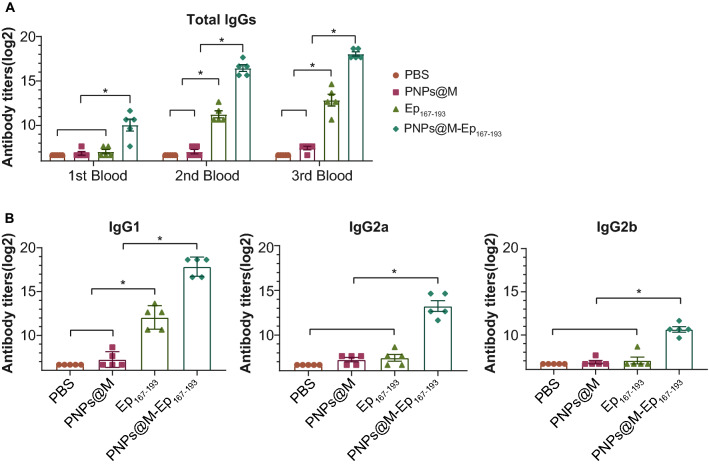
PNPs@M-Ep_167-193_ induced a Th2-biased immune response in mice. **A**. The bars represent the Ep_167-193_-specific IgG titers in the sera of immunized mice (n = 5) determined by ELISA 1 week after the first, second and final immunization, respectively. **B**. The subtypes of anti-Ep_167-193_ antibodies. The bars represent the titers of IgG1, IgG2a, and IgG2b against Ep_167-193_ 1 week after the last immunization. The data are shown as the mean ± SE. Multiple comparisons among the four groups were analyzed by one-way ANOVA. *P < 0.05

The level of Ep_167-193_-specific antibodies was determined seven days after each immunization. At seven days after the first immunization, Ep_167-193_-specific IgGs were detectable in the sera of PNPs@M-Ep_167-193_ group, while no significant increase was observed in the other three groups. After the second immunization, the Ep_167-193_-specific IgG titers were significantly increased in both PNPs@M-Ep_167-193_ and naked Ep_167-193_ group. And the titer of PNPs@M-Ep_167-193_ was higher than that of Ep_167-193_ alone (*P* < 0.05)_._ Similarly, after the third immunization the titer of anti-Ep_167-193_ IgGs from the PNPs@M-Ep_167-193_ group was also significantly higher than that of the naked Ep_167-193_ group (*P* < 0.05), despite of the limited increase in Ep_167-193_ group. However, no significant change in the antibody titers was observed in the PNPs@M and PBS control groups. Moreover, the IgG titers increased slightly after the third immunization. The trend among the four groups was consistent with the second immunization (Fig. 6A). Further analysis of the IgG subtypes revealed that PNPs@M-Ep_167-193_ immunization led to significantly elevated IgG1, IgG2a and IgG2b levels. Moreover, the increase in IgG1 was the most predominant (Fig. 6B). Collectively, these results suggested that PNPs@M-Ep_167-193_ induced a Th2-biased immune response in mice.

### PNPs@M-Ep_167-193_ immunization conferred effective protection in mice

**Fig. 7:**
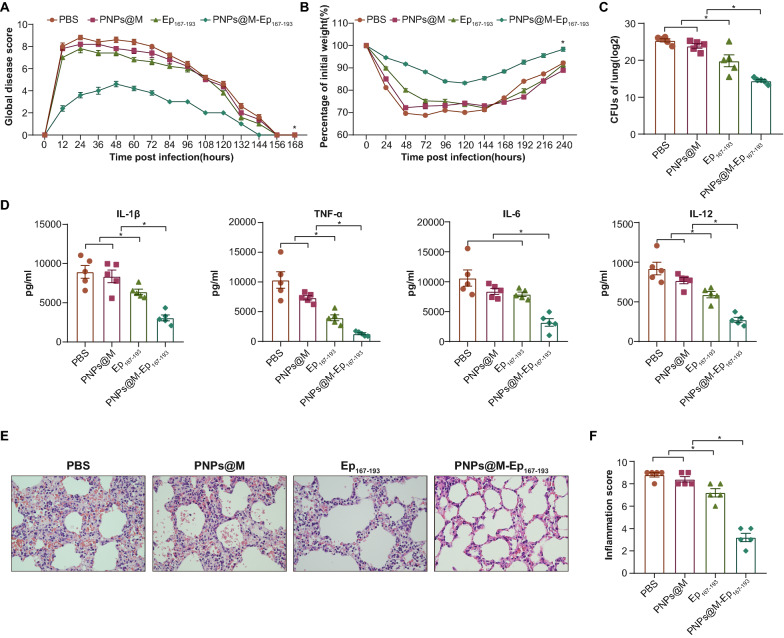
PNPs@M-Ep_167-193_ immunization conferred effective protection in mice. **A**. The global disease scores of the mice immunized with PNPs@M-Ep_167-193,_ Ep_167-193,_ PNPs@M or PBS after challenge with a sublethal dose of PA XN-1 (n = 5). Scores were recorded every 12 h for 7 days. **B**. Changes in mouse body weights after their immunization with PNPs@M-Ep_167-193,_ Ep_167-193,_ PNPs@M or PBS and challenge with a sublethal dose of PA XN-1 (n = 5); weights were recorded every 24 h for 10 days. **C**. Bacterial loads in the lungs were determined 24 h after the challenge with a sublethal dose of PA XN-1 (n = 5). The bar represents the average and SE of the log CFU of PA XN-1. Multiple comparisons among the four groups were analyzed by one-way ANOVA. *P < 0.05. D. Quantitative measurement of the proinflammatory cytokines IL-1β, TNF-α, IL-6 and IL-12 in the lungs (n = 5). The data are shown as the mean ± SE. Multiple comparisons among the four groups were analyzed by one-way ANOVA. *P < 0.05. **E**. HE staining images of the lungs from immunized mice 24 h after infection with a sublethal dose of PA XN-1. Images were captured at 400 × magnification. **F**. Semiquantitative analysis of lung inflammation. The bars represent the inflammation scores of the images according to hemorrhage, edema, hyperemia and neutrophil infiltration. Multiple comparisons among the four groups were analyzed by one-way ANOVA. *P < 0.05

We then evaluated the effect of PNPs@M-Ep_167-193_ on immunity elicited by intratracheal injection of PA XN-1. Figure 7A shows the disease scores of the mice within seven days after the challenge. The onset of infection symptoms in the PNPs@M-Ep_167-193_ group was clearly slower than that in the other groups and presented with limited magnitude. In addition, the mice in this group required less time to return to health. The AUC (area under the curve) of the disease score was also significantly smaller in the PNPs@M-Ep_167-193_-treated group than that in the other three groups. However, no significant difference was observed among the PBS, PNPs@M and Ep_167-193_ groups. The patterns of weight change and disease scores were very similar between these four groups (Fig. 7B). Figure 7C shows the number of bacteria colonized in the lung 24 h post challenge. The number of bacteria in the mice from the PNPs@M-Ep_167-193_ group was significantly lower than that in the Ep_167-193_, PNPs@M vector and PBS control groups of mice. Nevertheless, a significant reduction in bacterial load was observed in mice immunized with Ep_167-193_ alone, indicating a restricted protection.

Additionally, to further clarify lung inflammation, we measured the levels of the proinflammatory cytokines IL-1β, TNF-α, IL-6 and IL-12 in the lungs. The levels of these four cytokines in the mouse lungs from the PNPs@M-Ep_167-193_ group were significantly lower than those in the other three groups (Fig. 7D). In addition, significant reductions in the levels of the tested proinflammatory cytokines were also found in the Ep_167-193_ group, with the exception of IL-6. A similar trend was found after histopathological analysis of the lungs (Fig. 7E). Congestion and neutrophil infiltration in the lungs of the PNPs@M-Ep_167-193_-treated mice were significantly restricted. The alveolar structures were not severely damaged, showing significant differences compared with the other three groups. The results from the pathological scoring also validated these trends (Fig. 7F). These data suggest that PNPs@M-Ep_167-193_-induced immunity conferred effective protection in mice.

## Discussion

Current PA vaccines mainly focus on lipopolysaccharides, secreted toxins, flagellin and outer membrane proteins [33]. Nevertheless, outer membrane proteins have attracted more attention due to their following advantages. First, antibodies against outer membrane proteins can mediate ADCC effects. In addition, these antibodies are able to block the functions of the outer membrane proteins, such as cell adhesion, bacterial motility, nutrient uptake, and immune escape. However, the strong hydrophobicity of the transmembrane regions of outer membrane proteins severely compromises the applications of these proteins as candidate antigens. One solution is to find soluble fragments, such as that from the outer membrane protein OprF, a fragment of which was found to have good water solubility and immunogenicity [10]. In this study, we tried a different strategy to invent vaccines targeting outer membrane proteins. To date, we have structurally predicted the extracellular domains of PA transmembrane proteins and screened epitopes that conferred protection. Similar to our previous results [15], our data provide additional evidence for the feasibility and effectiveness of this strategy, identifying an alternative solution for other bacterial vaccines targeting transmembrane proteins.

A critical problem with epitope-based vaccines is the optimization of the delivery system, which is beneficial to improve the immunogenicity, stability and bioavailability of these vaccines [34]. Such delivery systems contain nanoemulsions, nanoparticles, VLPs, etc. In this study, PLGA nanoparticles wrapped in macrophage membranes were used to deliver Ep_167-193_, which generated the nanovaccine PNPs@M-Ep_167-193_. The results showed that PNPs@M-Ep_167-193_ is safe in vitro and in vivo. Moreover, PNPs@M-Ep_167-193_ was able to induce significantly a stronger Th2 immune response and confer adequate protection in mice. Our data showed that PNPs@M-Ep_167-193_ is approximately 272 nm and negatively charged and can be easily taken up by DC cells. In addition, the macrophage membrane on the surface of this nanovaccine benefits its safety and processing. These results suggest that PNPs@M is an efficient vector for epitope delivery and that PNPs@M-Ep_167-193_ is a promising candidate vaccine against PA.

One problem during the development of bacterial vaccines is that the expression of virulence genes varies over time throughout the infection process. If the expression of the vaccine target is reduced or even stopped, the protection conferred by vaccine would undoubtably be impaired. However, newly expressed or upregulated proteins could serve as ideal antigenic targets [35]. For example, PilA and PilQ are expressed during *Vibrio cholerae* infection and have been identified as promising vaccine candidates [36]. In the present study, we focused on the upregulated outer membrane proteins and identified the protective epitope Ep_167-193_ by Luminex combined with bioinformatics. Our results demonstrate once again the important contribution of in vivo-induced antigen technology in vaccine development.

In this study, we identified ten epitopes with good immunogenicity from eight outer membrane proteins using human sera. Interestingly, the immunogenicity of the epitopes PA0958 A200-Q235 and PA2398 F636-G672 was significantly reduced when tested in the mouse model. This may be due to the genetic differences between humans and mice. Another reason for this result could be that these synthetic peptides did not adopt the correct conformation (that of the native protein). However, these results do not mean that epitopes with weak immunogenicity in mice do not protect humans. In the future, the protective effects of these epitopes could be evaluated in HLA transgenic mice or human experiments.

In this study, we also found that the sera of some healthy volunteers reacted to synthesized epitope peptides; for example, PA2398 Q737-K754, PA2398 M684-N615, and PA1178 G32-T62. One reason for this reactivity may be that these volunteers had been infected with PA, and PA-specific antibodies persisted in the sera for a long time. Another possible reason is that these peptides share certain common epitopes with other pathogens, which leads to a cross-reaction. Therefore, we plan to collect samples from a larger cohort of healthy volunteers or sera from younger donors to address this issue.

PA4554 refers to the type IV pilus biogenesis factor PilY1. By interacting with PilVWX, PilY1 is involved in type IV pili assembly, twitching motility and adhesion to host cells [37]. Our finding that PA4554 was upregulated during PA infection provided additional evidence for the contribution of PA4554 in PA pathogenesis. Moreover, the fact that targeting the Ep region conferred immunoprotection against PA infection also showed the significance of this region. Therefore, further studies could focus on determining the exact structure and function of the Ep_167-193_ region of PA4554.

In summary, through the use of structural tools and in vivo-induced antigen technology, we identified the novel immunogenic epitope PA4554 Ep_167-193_ from PA. Moreover, we constructed a macrophage membrane-encapsulated PLGA nanoparticle vaccine carrying PA4554 Ep_167-193_, which elicits a Th2 immune response and confers adequate protection in mice.

## Supplementary Information

**Additional file 1: Table S1.** Relative expression levels of outer membrane proteins.

**Additional file 2: Table S2.** Amino acid sequence of 54 candidate peptides.

**Additional file 3: Table S3.** List of abbreviations.

**Additional file 4: Figure S1.** Prediction of transmembrane loops of the eight transmembrane proteins (PA1178, PA1777, PA4067, PA4554, PA0595, PA0958, PA2398, PA0165) by PRED-TMBB software. Extracellular sequence, transmembrane sequence and intracellular sequence were shown in blue, red and green, respectively.

**Additional file 5: Figure S2.** Structure validation of PA1178, PA4554 and PA0165 by Procheck and QMEAN.

**Additional file 6: Figure S3.** TEM images of PNPs (left) and PNPs@M (right). The scale is 200 nm.

**Additional file 7: Figure S4.** Toxicity evaluation of PNPs@M-Ep_167-193_ and PNPs@M. **A**, **B** The survival of DC2.4 cells and L929 cells exposed to different concentrations of PNPs@M-Ep_167-193_ for 48 h (**A**) and 72 h (**B**). **C**–**E** The survival of DC2.4 cells and L929 cells exposed to different concentrations of PNPs@M for 24 h (**C**), 48 h (**D**) and 72 h (**E**).

**Additional file 8: Figure S5.** Toxicity evaluation of PNPs@M-Ep_167-193_. Percentage of initial body weight (**A**) and body temperature (**B**) during the 35 days of observation (n=5).

## Data Availability

The datasets used and/or analyzed during the current study are available from the corresponding author on reasonable request.
