# Supplementary material for: A novel structurally identified epitope delivered by macrophage membrane-coated PLGA nanoparticles elicits protection against Pseudomonas aeruginosa
Source: J Nanobiotechnology. 2022 Dec 14;20:532. doi: 10.1186/s12951-022-01725-x (PMC9750051; doi:10.1186/s12951-022-01725-x)
Supplement: Supplementary file 4 — Additional file 4: Figure S1. Prediction of transmembrane loops of the eight transmembrane proteins (PA1178, PA1777, PA4067, PA4554, PA0595, PA0958, PA2398, PA0165) by PRED-TMBB software. Extracellular sequence, transmembrane sequence and intracellular sequence were shown in blue, red and green, respectively. [file 12951_2022_1725_MOESM4_ESM.pdf]

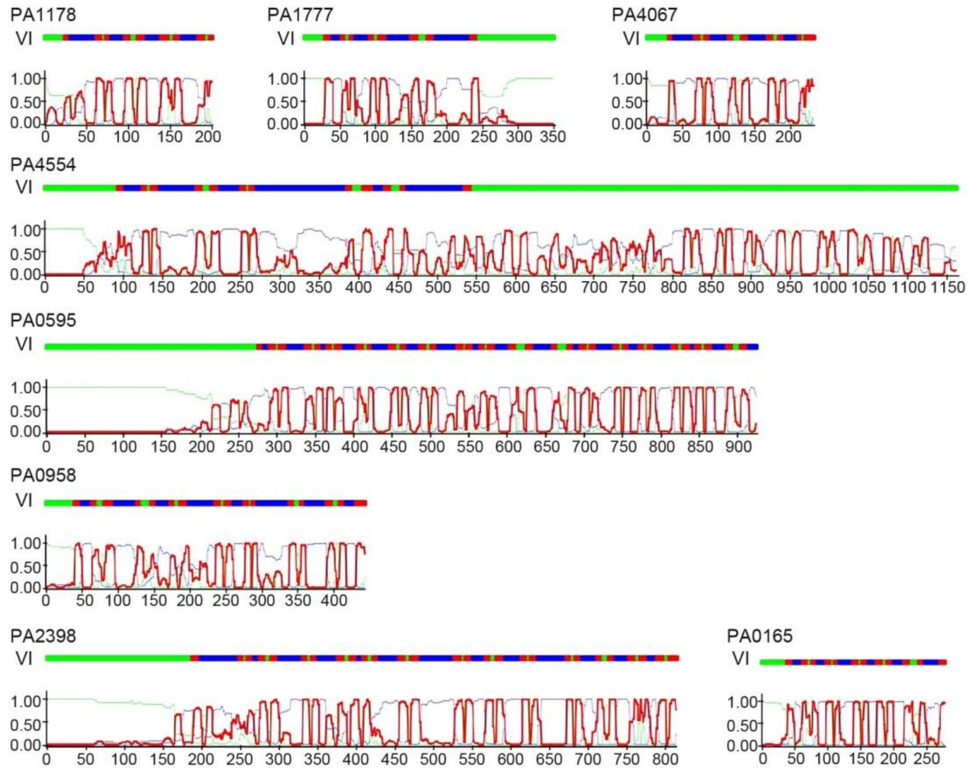

Figure S1. Prediction of transmembrane loops of the eight transmembrane proteins (PA1178, PA1777, PA4067, PA4554, PA0595, PA0958, PA2398, PA0165) by PRED-TMBB software. Extracellular sequence, transmembrane sequence and Intracellular sequence were shown in blue, red and green, respectively.
