# Supplementary figures and images for: A novel structurally identified epitope delivered by macrophage membrane-coated PLGA nanoparticles elicits protection against Pseudomonas aeruginosa
Source: J Nanobiotechnology. 2022 Dec 14;20:532. doi: 10.1186/s12951-022-01725-x (PMC9750051; doi:10.1186/s12951-022-01725-x)

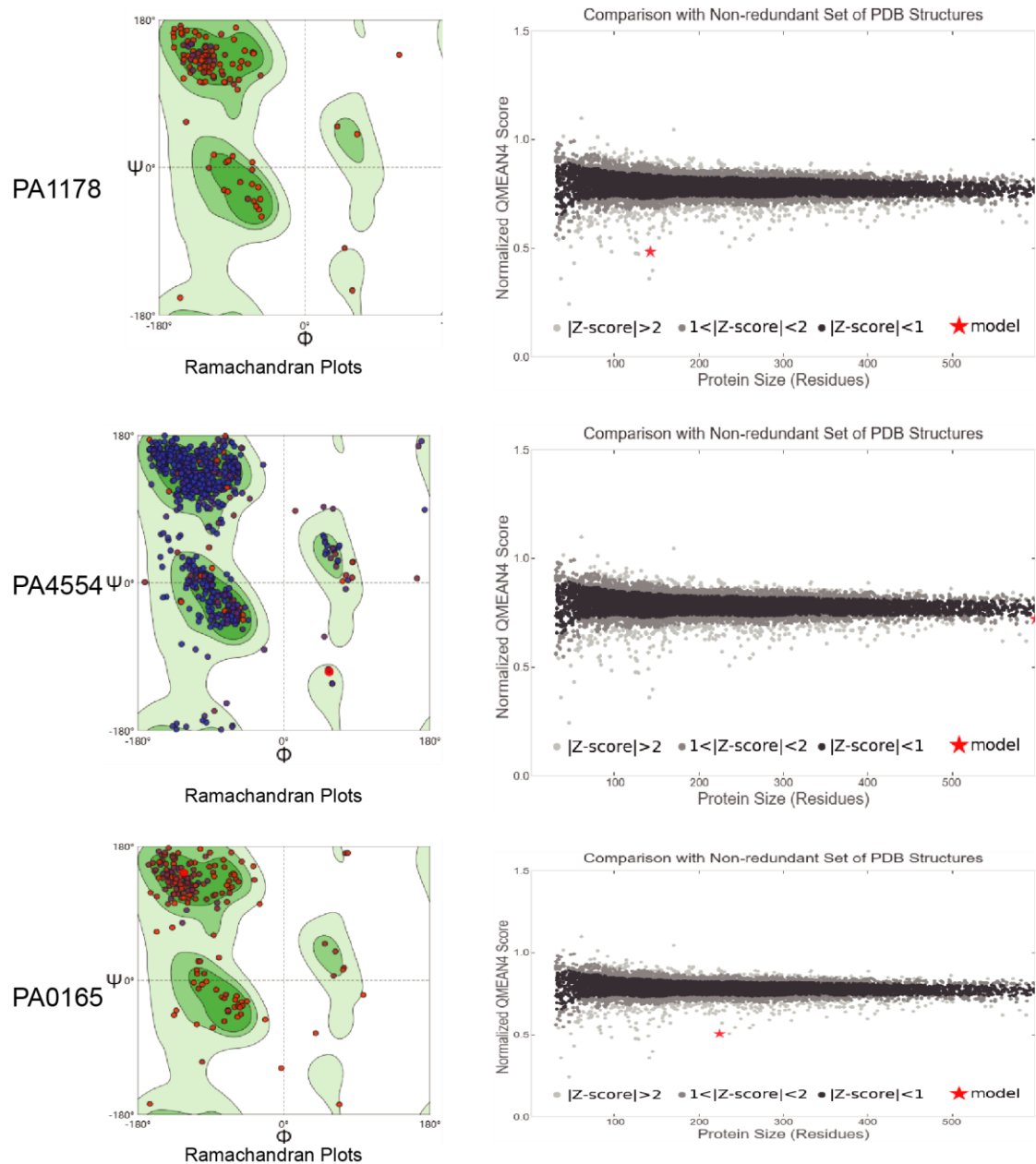

Figure S2. Structure validation of PA1178, PA4554 and PA0165 by Procheck and QMEAN.

Supplement: Supplementary file 5 — Additional file 5: Figure S2. Structure validation of PA1178, PA4554 and PA0165 by Procheck and QMEAN. [file 12951_2022_1725_MOESM5_ESM.pdf]

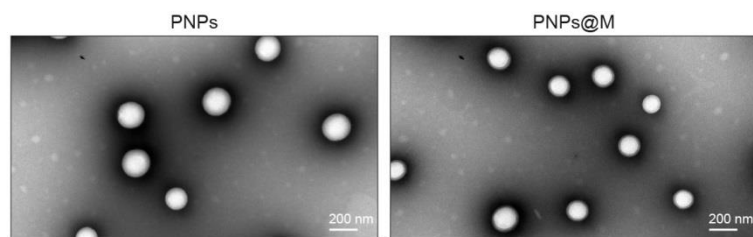

Figure S3. TEM images of PNPs (left) and PNPs@M (right). The scale is 200 nm.

Supplement: Supplementary file 6 — Additional file 6: Figure S3. TEM images of PNPs (left) and PNPs@M (right). The scale is 200 nm. [file 12951_2022_1725_MOESM6_ESM.pdf]
