# Supplementary material for: A novel structurally identified epitope delivered by macrophage membrane-coated PLGA nanoparticles elicits protection against Pseudomonas aeruginosa
Source: J Nanobiotechnology. 2022 Dec 14;20:532. doi: 10.1186/s12951-022-01725-x (PMC9750051; doi:10.1186/s12951-022-01725-x)
Supplement: Supplementary file 7 — Additional file 7: Figure S4. Toxicity evaluation of PNPs@M-Ep167-193 and PNPs@M. A, B The survival of DC2.4 cells and L929 cells exposed to different concentrations of PNPs@M-Ep167-193 for 48 h (A) and 72 h (B). C–E The survival of DC2.4 cells and L929 cells exposed to different concentrations of PNPs@M for 24 h (C), 48 h (D) and 72 h (E). [file 12951_2022_1725_MOESM7_ESM.pdf]

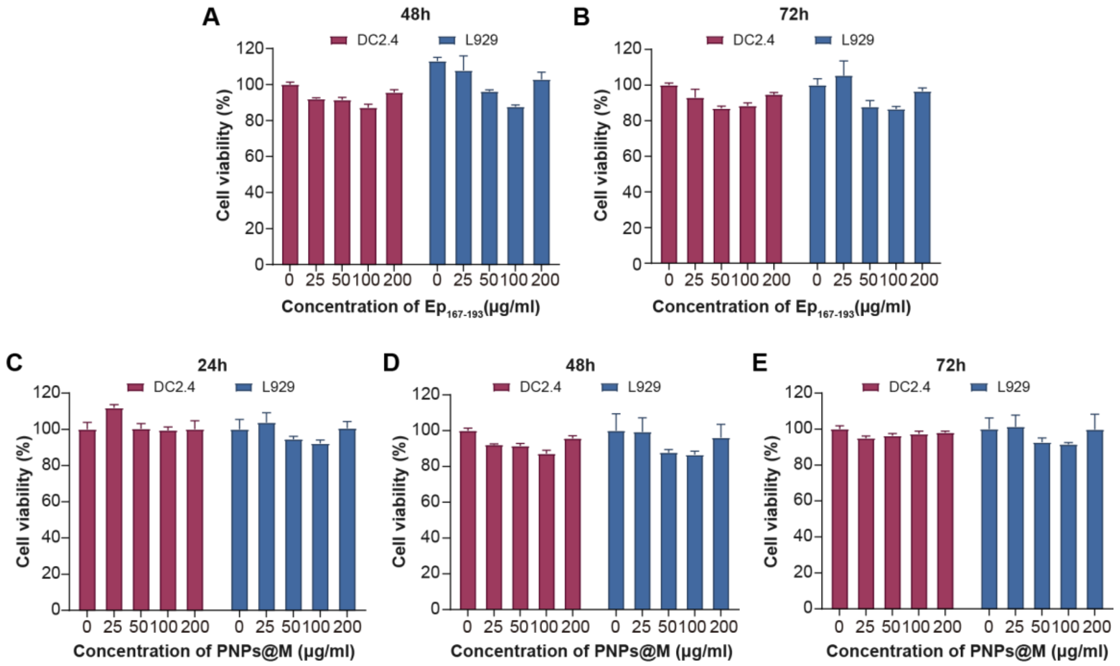

Figure S4. Toxicity evaluation of PNPs@M-$Ep_{167-193}$ and PNPs@M. A,B The survival of DC2.4 cells and L929 cells exposed to different concentrations of PNPs@M-$Ep_{167-193}$ for 48 h(A) and 72h (B). C-E The survival of DC2.4 cells and L929 cells exposed to different concentrations of PNPs@M for 24h(C),48 h(D) and 72h (E)
