# Supplementary material for: A novel structurally identified epitope delivered by macrophage membrane-coated PLGA nanoparticles elicits protection against Pseudomonas aeruginosa
Source: J Nanobiotechnology. 2022 Dec 14;20:532. doi: 10.1186/s12951-022-01725-x (PMC9750051; doi:10.1186/s12951-022-01725-x)
Supplement: Supplementary file 8 — Additional file 8: Figure S5. Toxicity evaluation of PNPs@M-Ep167-193. Percentage of initial body weight (A) and body temperature (B) during the 35 days of observation (n=5). [file 12951_2022_1725_MOESM8_ESM.pdf]

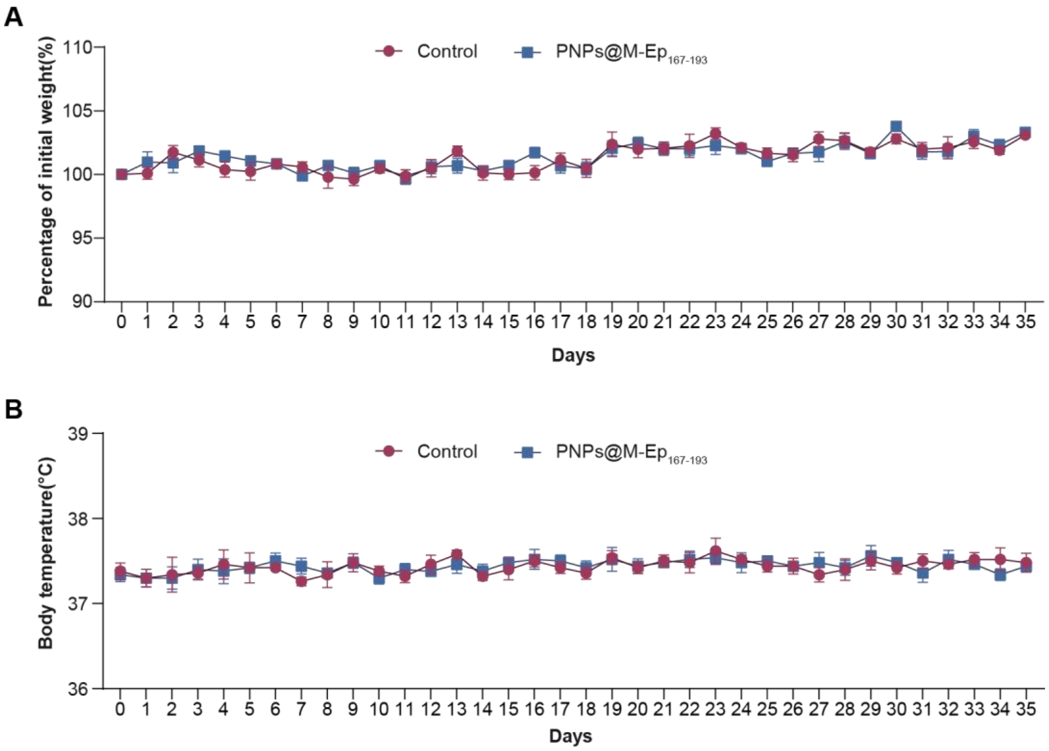

Figure S5. Toxicity evaluation of PNPs@M-Ep167-193. Percentage of initial body weight(A) and body temperature(B) during the 35 days of observation(n=5).
